# Determinants of contraceptive use among married women in Indonesia

**DOI:** 10.12688/f1000research.22482.1

**Published:** 2020-03-18

**Authors:** Alfian Gafar, Dewi Elizadiani Suza, Ferry Efendi, Eka Mishbahatul Mar’ah Has, Ahmad Putro Pramono, Ika Adelia Susanti

**Affiliations:** 1 Faculty of Nursing, Universitas Airlangga, Surabaya, Indonesia; 2 Faculty of Nursing, Universitas Sumatera Utara, Sumatera Utara, Indonesia; 3 School of Nursing & Midwifery, La Trobe University, Melbourne, Australia

**Keywords:** Contraceptive, Demographic and Health Survey (DHS), Determinants, Married Women

## Abstract

**Background**: Contraceptives in family planning are used to control the timings between pregnancies. Although the number of those using family planning has increased, determinants of contraceptive use among married women in Indonesia remain insufficient. This research aimed to identify the factors associated with contraceptive use among married women in Indonesia.

**Methods: **This study employed data from the Indonesian Demographic and Health Survey 2017. We selected 35,621 married women aged 15–49 years. Then, the determinants of contraceptive use among married women in Indonesia were examined by binary logistic regression.

**Results: **Women’s age (Adjusted Odds Ratio (AOR)=0.529; 95% CI=0.470–0.597), the number of living children (AOR=44.024; 95% CI=33.193–58.390), education level (adjusted odds ratio=2.800; 95% CI=2.181–3.594), wealth index (AOR=1.104; 95% CI=0.978–1.246), frequency of watching television (AOR=1.555; 95% CI=1.321–1.829), and frequency of using the Internet (AOR=0.856; 95% CI=0.794–0.924) were significantly associated with contraceptive use among married women.

**Conclusions: **This study highlights the determinants of contraceptive use among married women in Indonesia. Women’s age, the number of living children, education level, wealth index, and access to information may influence contraceptive use among these women. This study emphasizes that health education and promotion on the importance of using contraception should be initiated in innovative ways.

## Introduction

Family planning is a conscious effort made by couples to limit the number of children through the use of contraceptive methods. Both developed and developing countries worldwide have demonstrated increased contraceptive use to control the population ^1^. Though contraceptives have been used globally, they remain less prevalent in poorer countries ^2^. In Indonesia, the prevalence of contraceptive use among married women is still low and varies between provinces, economic status, education level, and residential location ^3^. Based on data from the Indonesian health ministry, the percentage of contraception is divided into the following four categories: 59.3% of married women aged 15–49 years use modern contraception methods (implants, tubectomy, vasectomy, intrauterine devices, condoms, injections, and pills), 0.4% use traditional methods (lactation amenorrhea methods, periodic abstinence, and interrupted into intercourse), 24.7% have done family planning at least once, and 15.5% have never done family planning ^3– 5^. Although the number of those who actively use family planning has increased, it has not been able to sustain or increase the prevalence of contraceptive use ^6^.

National data show that contraceptive use fell from 61.75% in 2014 to 59.98% in 2015 ^6^. One of the challenges in increasing the number of family planning participants is the high level of concern from couples of reproductive age regarding the side effects of contraceptive drugs and equipment ^6^. Researchers in Ghana have performed a study to identify the factors related to the low contraception use. They found that such factors included the residential location, knowledge, marital status, religion, and partner’s approval and support ^7^. All efforts that can improve women’s health should be implemented, particularly regarding maternal healthcare services ^8^. Thus, the current study aimed to identify factors that are associated with contraceptive use among married women in Indonesia.

## Methods

### Data

The data for this study were collected from the 2017 Indonesia Demographic and Health Survey (DHS), which is the eighth survey since 1987. The IDHS in 2017 was performed with the cooperation of the Central Statistics Agency, the National Population and Family Planning Agency, and the Ministry of Health, with technical assistance from the Inner City Fund (ICF) internationally through the Demographic Project and Health Surveys (DHS) Program. We used the individual recoded dataset in this study.

### Sample size and sampling

Our sample was composed of married women aged 15–49 years who were using contraception in Indonesia. The survey successfully interviewed 49,627 women. Based on the inclusion criteria, 35,621 women remained. The IDHS used two-stage stratified cluster sampling to select the sample, including 1,970 census blocks covering urban and rural areas. The inclusion criteria in this study were all married women aged 15–49 years and who answered the questionnaire properly, whereas the exclusion criterion was women who were not married.

### Variables

The dependent variable of this study was contraceptive use, which was defined in this study as the use of contraception by married women at the time of the survey. For the explanatory variables, we used the women’s age, the number of living children, education level, wealth index, residential location, the frequency of watching television, and access to the Internet.

### Data analysis

The data were analyzed using the STATA statistical software version 14. Before the analysis, the dataset was weighted to account for any differences, considering the nature of the sampling design method. Univariate analysis and bivariate analysis were performed using the chi-square test. Finally, the determinants of contraceptive use among married women were identified using the binary logistic regression.

**Ethical review and consent**

The IDHS in 2017 obtained ethical permits from the Ministry of Health of Indonesia. All respondent identifiers were deleted from the data, and written informed consents were provided by each participant. The ICF International, which is part of the DHS program, approved the use of such data in this study.

## Results

More than half of the respondents used contraception (63.60%) in which majority of them aged 35–49 years (54.77%). According to the data, contraceptive use was most prevalent in married women who had 1–2 children (62.58%). Furthermore, most of the respondents had completed secondary education (52.03%) and were classified as richer by the wealth index (21.22%). Meanwhile, slightly more than half of them were from rural areas (51.62%). Regarding information access, 34,367 (96.48%) relied on watching television, whereas 12,733 (35.74%) relied on the Internet. Data are presented in Table 1.

**Table 1.** Socio–demographic characteristics of the Indonesian women.

Variable	N	%
**Contraception** Use Not use	22,655 12,966	63.60 36.40
**Women age** 15–24 years	4,011	11.26
25–34 years	12,101	33.97
35–49 years	19,509	54.77
**Number of living children** No children	2,698	7.57
1–2	22,293	62.58
3–4	9,108	25.57
5+	1,522	4.27
**Education** No education	661	1.86
Primary	12,061	33.86
Secondary	18,533	52.03
Higher	4,366	12.26
**Wealth index** Poorest	6,287	17.65
Poorer	7,094	19.91
Middle	7,380	20.72
Richer	7,560	21.22
Richest	7,300	20.49
**Place of residence** Urban	17,234	48.38
Rural	18,387	51.62
**Access to television** Never	1,254	3.52
Yes	34,367	96.48
**Access to the internet** Never	22,888	64.26
Yes	12,733	35.74

In the bivariate analysis, all of the variables, except for the residential location, were significantly associated with contraceptive use among married women ( Table 2).

**Table 2.** Bivariate analysis of the determinants of contraceptive use among married women in Indonesia.

Variables	Contraception				*X* ^2^
	Use		Not use		
	n	%	n	%	
**Women age**					
15–24 years	2,283	10.08	1,728	13.33	84.6835 ***
25–34 years	7,810	34.47	4,291	33.09	
35–49 years	12,563	55.45	6,946	53.58	
**Number of** **living children**					
No children	126	0.55	2,572	19.84	4410.2272 ***
1–2	14,940	65.95	7,353	56.71	
3–4	6,697	29.56	2,411	18.59	
5+	892	3.94	630	4.86	
**Education**					
No education	244	1.08	417	3.22	311.4141 ***
Primary	7,937	35.03	4,125	31.81	
Secondary	11,990	52.92	6,543	50.46	
Higher	2,484	10.97	1,881	14.51	
**Wealth index**					
Poorest	3,820	16.86	2,466	19.02	60.7842 ***
Poorer	4,695	20.72	2,399	18.50	
Middle	4,838	21.36	2,542	19.61	
Richer	4,773	21.07	2,787	21.49	
Richest	4,529	19.99	2,772	21.38	
**Place of** **residence**					
Rural	11,798	52.08	6,589	50.82	5.0720
Urban	10,857	47.92	6,377	49.18	
**Access to** **television**					
No	634	2.8	620	4.78	91.8526 ***
Yes	22,021	97.2	12,346	95.22	
**Access to the** **internet**					
No	15,076	66.55	7,812	60.25	137.3068 ***
Yes	7,579	33.45	5,154	39.75	

*p-value<0.05; **p-value<0.01: ***p-value<0.001

In the multivariate analysis, the association between the independent and dependent variables was assessed by binary logistic regression ( Table 3). Married women aged 35–49 years were less likely to use contraceptives than married women aged 15–24 years (Adjusted Odds Ratio (AOR) =0.529; 95% CI=0.470–0.597). Furthermore, married women with more than five children were more likely to use contraception than those without children (AOR=44.024; 95% CI=33.193–58.390). Women who had completed higher education had 2.8 times greater odds of using contraceptives (AOR=2.800; 95% CI=2.181–3.594) than those who had not completed any formal education. Women classified as the richest by the wealth index were 1.1 times more likely to use contraceptives than those classified as poorest (AOR=1.104; 95% CI=0.978–1.246). Women who watched television were more likely to use contraceptives (AOR=1.555; 95% CI=1.321–1.829) than those who never watched television. In comparison, married women who accessed information from the Internet were less likely to use contraceptives (AOR=0.856; 95% CI=0.794–0.924).

**Table 3.** Binary logistic regression of the determinants of contraceptive use among married women in Indonesia.

Variables	AOR	95% CI	
		Lower	Upper
**Age**			
15–24 years	Ref		
25–34 years	0.666 ***	0.592	0.749
35–49 years	0.529 ***	0.470	0.597
**Number of living children**			
No children	Ref		
1–2	48.873 ***	37.961	62.921
3–4	76.743 ***	59.015	99.796
5+	44.024 ***	33.193	58.390
**Education**			
No education	Ref		
Primary	2.833 ***	2.246	3.572
Secondary	2.969 ***	2.351	3.750
Higher	2.800 ***	2.181	3.594
**Wealth index**			
Poorest	Ref		
Poorer	1.198 ***	1.082	1.326
Middle	1.174 ***	1.054	1.309
Richer	1.090 ***	0.977	1.216
Richest	1.104 ***	0.978	1.246
**Access to television**			
Never	Ref		
Yes	1.555 ***	1.321	1.829
**Access to the internet**			
Never	Ref		
Yes	0.856 ***	0.794	0.924

^*^p-value<0.05; **p-value<0.01: ***p-value<0.001

## Discussion

This study sought to assess the determinants of contraceptive use among married women in Indonesia. We first found that women’s age, especially older age, was significantly associated with contraceptive use among married women. This result is consistent with the previous studies conducted in Ghana and Nigeria, documenting that older women have a lower level of concern with modern contraceptive use ^7, 9^. This finding is related to their lower fecundity rates and less active sexual desires ^10^. The benefits of using contraception are to delay or space subsequent pregnancies and to limit the number of children ^2, 11, 12^. Although older women use contraception less often than young women, they still considered the use of contraceptives; of note, only a small proportion of women aged 35–49 reported having gone through menopause.

Women with five or more living children were more likely to use contraceptives. This finding is consistent with those of studies performed in Ghana ^7, 13^. The addition of one child will increase the tendency of married women to use contraception by 7%–8%. Another study also found that women with more than three living children were more likely to use contraceptives than those without children, and their reason of using contraceptives was to prevent from having more children ^9, 10, 14, 15^. Women will choose to use contraception when they have reached their ideal family size ^7^. Therefore, women who have many children are more likely to use contraception because they are more likely to have reached their ideal family size. The World Health Organization has reported that maternal mortality is increased in women who have more than four children ^1^. Multiparous women have the highest risk of maternal death; therefore, contraceptive use should be encouraged to reduce this mortality ^16^.

Moreover, education levels were significantly associated with contraceptive use among these married women. Higher education levels correlated with higher use of contraceptives. Two previous studies conducted in Bangladesh and Ghana reported that education has an extremely significant influence on contraceptive use. In this report, women with higher education were more likely to use contraception than those without formal education ^14, 15^. This finding is the result of highly educated people being more likely to be aware of the benefits and importance of using contraception ^14^. Another study in Nigeria also found that educated women were more likely to use contraceptives ^9^. Education is commonly assumed by someone’s knowledge. Someone with higher education level has better knowledge. Education remains an important factor in terms of increasing women’s knowledge regarding family planning.

Wealth index was also significantly associated with contraceptive use among these married women. Women from the richest tier of the wealth index also had odds that progressively increased to using a contraceptive. Similar findings regarding the strong association between wealth index and contraceptive use were obtained by studies conducted in Malawi and Ghana; women classified within the richest wealth index were more likely to use contraception than those grouped in the poorest index ^7, 9, 15, 17^. In 2017, Ofonime stated that financial factors play an important role in the decreased use of contraceptives among the poorest married women ^18^. Providing free access for contraceptives to poor women would be beneficial to increase the use of contraceptives.

Meanwhile, access to information showed a significant association with contraceptive use among these married women. Mubashar *et al.* in 2016 found that most sources of contraception information were from media, such as television, radio, the Internet, local news, newspaper, and magazine ^19^. Access to information from television was significantly associated with contraceptive use. Women who watched television had approximately 1.5 greater odds of using contraceptives than those who never watch television. This finding is consistent with the previous study conducted in Ethiopia and Ghana ^20– 22^. Exposure to media, such as television, is an important factor related to women’s knowledge about contraceptive use ^20^. Watching television is important to increase the knowledge of the wider community to understand the types, benefits, and methods of using contraception correctly. In contrast, this study also showed that access to information via the Internet was significantly associated with less likelihood of contraceptive use among married women. This finding is supported by a previous study conducted in Australia ^23^. In such study, 50% of women reported that they felt dissatisfied with the quality and quantity of information about contraception, especially oral contraceptive pill, they obtained through the Internet. Improving the sources of information about contraceptives for married women will be the best approach to encourage contraceptive use and avoid bias information from unreliable media sources.

## Conclusions

Factors such as women’s age, the number of living children, education level, wealth index, and access to information remain significant issues in determining contraceptive use among married women in Indonesia. Overall, the study results suggest that policymakers should target certain women and create a campaign regarding contraception. Targeting older, poor, and uneducated or less educated women may have a positive impact in terms of increasing their use of contraception.

## Data availability

### Source data

Data used in this study is available online from the Indonesian 2017 Demographic and Health Survey (DHS) website under the ‘Individual Recode’ section.

Data can be accessed by applying through the DHS website. Please see their data access help page for information.
