# Supplementary material for: Determinants of contraceptive use among married women in Indonesia
Source: F1000Res. 2020 Mar 18;9:193. [Version 1] doi: 10.12688/f1000research.22482.1 (PMC7137393; doi:10.12688/f1000research.22482.1)
Supplement: Peer review history

# Peer review: Reviewer response for version 1

**DOI:** 10.5256/f1000research.24812.r61505

**Published:** 2020-04-06

**Authors:** Khatijah Binti Abdullah Lim Geok Khim

**Affiliations:** 1 Department of Nursing Sciences, Faculty of Medicine, University of Malaya, Kuala Lumpur, Malaysia

This manuscript is well written with clear explanation of the methodology used. Data collected were analysed using appropriate statistical tests. Interpretations of the data were correctly displayed and discussed. Study findings highlight the determinants of contraceptive use among married women in Indonesia which can help in formulating relevant policy and strategies in improving contraceptive use in Indonesia. I have no recommendation for any revision to the manuscript in its current format and have no objection for it to be indexed with no revision.

I confirm that I have read this submission and believe that I have an appropriate level of expertise to confirm that it is of an acceptable scientific standard.

# Peer review: Reviewer response for version 1

**DOI:** 10.5256/f1000research.24812.r61507

**Published:** 2020-03-24

**Authors:** Angeline Bushy

**Affiliations:** 1 College of Nursing, University of Central Florida, Orlando, FL, USA

This manuscript is cogently organized with an appropriate quantitative methodology. The findings can provide insights to educators, researchers, as well as policymakers regarding family planning education and contraceptive needs and developing countries. I do not offer any editorial suggestions or revisions to the current manuscript. I recommend indexing without revision.

I confirm that I have read this submission and believe that I have an appropriate level of expertise to confirm that it is of an acceptable scientific standard.
